# RDA coupled with deep sequencing detects somatic SVA-retrotranspositions and mosaicism in the human brain

**DOI:** 10.3389/fcell.2023.1201258

**Published:** 2023-06-01

**Authors:** Jonas Möhner, Maurice Scheuren, Valentina Woronzow, Sven Schumann, Hans Zischler

**Affiliations:** ^1^ Division of Anthropology, Institute of Organismic and Molecular Evolution, Faculty of Biology, Johannes Gutenberg University Mainz, Mainz, Germany; ^2^ Institute of Anatomy, University Medical Center of the Johannes Gutenberg University Mainz, Mainz, Germany

**Keywords:** SINE-VNTR-Alu, retrotransposon, somatic mosaic, human brain, representational difference analysis

## Abstract

Cells of the developing human brain are affected by the progressive acquisition of genetic and epigenetic alterations that have been reported to contribute to somatic mosaicism in the adult brain and are increasingly considered a possible cause of neurogenetic disorders. A recent work uncovered that the copy–paste transposable element (TE) LINE-1 (L1) is mobilized during brain development, and thus mobile non-autonomous TEs like AluY and SINE-VNTR-Alu (SVA) families can use L1 activity in trans, leading to *de novo* insertions that may influence the variability of neural cells at genetic and epigenetic levels. In contrast to SNPs and when considering substitutional sequence evolution, the presence or absence of TEs at orthologous loci represents highly informative clade markers that provide insights into the lineage relationships between neural cells and how the nervous system evolves in health and disease. SVAs, as the ‘youngest’ class of hominoid-specific retrotransposons preferentially found in gene- and GC-rich regions, are thought to differentially co-regulate nearby genes and exhibit a high mobility in the human germline. Therefore, we determined whether this is reflected in the somatic brain and used a subtractive and kinetic enrichment technique called representational difference analysis (RDA) coupled with deep sequencing to compare different brain regions with respect to *de novo* SINE-VNTR-Alu insertion patterns. As a result, we detected somatic *de novo* SVA integrations in all human brain regions analyzed, and the majority of *de novo* insertions can be attributed to lineages of telencephalon and metencephalon, since most of the examined integrations are unique to different brain regions under scrutiny. The SVA positions were used as presence/absence markers, forming informative sites that allowed us to create a maximum parsimony phylogeny of brain regions. Our results largely recapitulated the generally accepted evo-devo patterns and revealed chromosome-wide rates of *de novo* SVA reintegration targets and preferences for specific genomic regions, e.g., GC- and TE-rich regions as well as close proximity to genes that tend to fall into neural-specific Gene Ontology pathways. We concluded that *de novo* SVA insertions occur in the germline and somatic brain cells at similar target regions, suggesting that similar retrotransposition modes are effective in the germline and soma.

## 1 Introduction

To date, the origin or the genetic and regulatory-epigenetic mechanisms by which the enormous amount of morphological and functional variability of somatic—here mainly neural—cells is generated remains poorly understood. Several experimental analyses have shown that cells of the brain differentially exhibit somatic genomic variation in a brain region-specific manner, which is partly associated with *de novo* retrotranspositions of transposable elements (TEs). Somatic mutations can be used to study the patterns of progenitor proliferation, migration, and differentiation underlying brain developmental processes. To this end, high-throughput sequencing has been performed to determine single-nucleotide variants (SNVs) (Lodato et al., 2015), whereby position-specific mutation rates, resulting in reversals, possibly create interpretational difficulties in the evaluation of mosaicism. On the other hand, an undisputable character polarity is associated with the retrotransposition of mobile elements. These elements amplify and colonize metazoan genomes by a germline ‘copy-and-paste’ mechanism associated with different activities of long interspersed element-1 (LINE-1 or L1). However, LINE-1 is the only active autonomous retroelement in the human genome, and non-autonomous elements rely on the enzymatic machinery provided by L1 for retrotransposition. Insertional mutagenesis and disease are linked with three families, namely, L1, Alu, and SINE-VNTR-Alu (SVA), all of which rely on ‘copy-and-paste’ mechanisms (reviewed by Cordaux and Batzer, 2009).

L1 expression in the human brain suggests that L1 mobilization may also occur during later development, and this assumption was tested with several NGS-based sequencing strategies such as retroposon capture and comparing the germline with the hippocampus and caudate nucleus (Baillie et al., 2011) and single-cell WGS of neurons (Evrony et al., 2015). Concordantly, somatic insertions of L1, Alu, and SVA sequences were found in different comparative settings and brain regions. In contrast, the absolute rates of somatic LINE-1 element retrotransposition in the brain have been discussed intensively. Moreover, it was suggested that there were brain region-specific rates of mobility.

In the context of hominoid brain evolution, SVAs are of special interest, mainly because they represent the ‘youngest’ class of hominoid-specific retrotransposons. SVAs are comprised of a characteristic (CCCTCT)_n_ hexamer repeat, Alu-like region, variable number of tandem repeats (VNTRs), and the env-gene plus 3′-LTR from HERV-K10 (Wang et al., 2005; Cordaux and Batzer, 2009). SVAs are preferentially found in gene- and GC-rich regions and are thus hypothesized to differentially co-regulate nearby genes (Savage et al., 2013; Gianfrancesco et al., 2019; Barnada et al., 2022).

**FIGURE 1:**
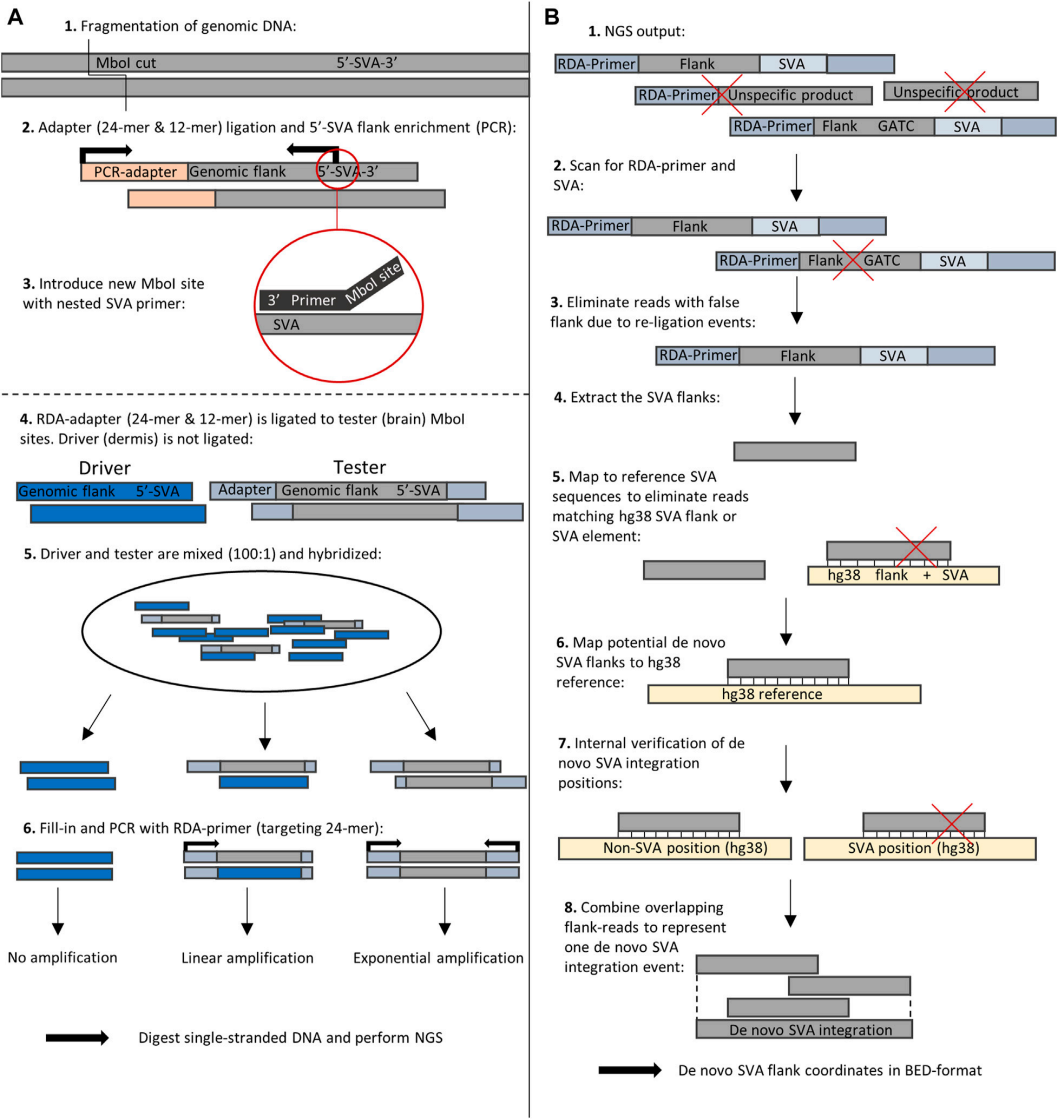
**(A)** SVA–RDA workflow. **(A1)** Genomic DNA was fragmented using MboI. Fragments were ligated with the “GATC”-ligatable PCR-adapter (double-strand consisting of a 24- and 12-mer sequence with sticky ends). **(A2)** 5′-SVA flanking regions were amplified by PCR. **(A3)** A second MboI site was introduced by nested PCR primers targeting the SVA region. **(A4)** After removing PCR-adapters using MboI, the “GATC”-ligatable RDA-adapter (double-strand consisting of a 24- and 12-mer sequence with sticky ends) can be ligated to both ends of the PCR products of the tester sample, while the driver sample remains untreated and does not contain adapters. **(A5)** The denatured and hybridized driver and tester samples were mixed at a ratio of 100:1, resulting in different double-stranded fragments that show different kinetic enrichment activities during PCR depending on the degree of RDA-adapter association. The RDA-primer targets the 24-mer sequence of the RDA-adapter, meaning fragment ends linked to the 12-mer sequence cannot be annealed. Unique fragments in the tester compared to the driver are enriched exponentially. This is a modified illustration based on the schematic representation (Figure 1) of Lisitsyn et al., (1993). **(B)** Bioinformatical analysis of RDA-SVA NGS data. **(B1,B2)** Only reads containing the RDA-primer and SVA sequence were extracted and **(B3)** reads containing an MboI site within the SVA flank were eliminated. **(B4)** The RDA-primer and SVA sequences were cut off to retrieve the SVA flank. **(B5)** The cleaned reads were mapped to reference SVAs and associated flanking sequences to exclude germline-fixed positions. **(B6)** The resulting reads were mapped to the human reference genome to identify *de novo* SVA integration positions. **(B7)** The positions were checked for re-alignments to known SVAs or SVA flanks as the internal verification step. **(B8)** The filtered *de novo* SVA flanks that share coordinates are accepted as one SVA integration event and combined into one chromosomal coordinate.

To define the genomic patterns of somatic *de novo* SVA integrations for different brain regions, we used a subtractive and kinetic enrichment technique coupled with deep sequencing to compare complex somatic genomes (Figure 1A). This method was introduced by Lisitsyn et al. (1993) and is termed representational difference analysis (RDA). Our approach was to specifically amplify the 5′-flanking region of SVAs by using ectodermal DNA from skin as a driver and comparing it with five different brain regions of two adult male donors as testers. To this end, MboI-restricted DNA is ligated with a “GATC”-ligatable adapter in driver and tester samples. The 5′-flanking SVA regions of interest are specifically enriched by PCR with an outward primer system as proposed in a mobile element scanning method for SVA (Ha et al., 2016). The driver PCR products are hybridized with the tester PCR products after ligation of RDA primers exclusively to the tester samples. During hybridization, three possible scenarios can occur: 1) both single strands are of driver origin, thus do not contain the tester-specific adapter, 2) a hybrid of tester-strand and driver-strand is generated and possesses one strand with the tester-derived adapter, and 3) both strands are of tester origin and contain the tester-derived adapters. After hybridization, a PCR reaction with primers specific to tester adapters can be performed. Driver–driver fragments without the adapter structure are not amplified, tester–driver fragments with one-sided adapter marking are linearly amplified and do not represent a unique transposon event in the tester (brain), and lastly tester–tester fragments with a *de novo* transposon insertion event are flanked by the RDA adapter on both sides and consequently amplified exponentially. Accordingly, if somatic retrotranspositions have occurred in the brain, the flank of the newly inserted SVA changes compared to that of skin ectodermal DNA and can be enriched and deep sequenced to estimate the full diversity of the heterogeneous PCR products. The NGS reads were then scanned for RDA primers, and a bioinformatics pipeline (Figure 1B) was developed to distinguish between hg38-annotated germline-transmitted SVAs and newly formed somatic SVA retrotranspositions in the olfactory bulb, cerebellum, prefrontal cortex, calcarine sulcus, and hippocampus. Moreover, to obtain an idea about the frequency of somatic *de novo* insertions of SVA, we took advantage of the well-defined character polarity of SVA insertions that allows each somatic insertion to be traced to a unique molecular event in a common ancestor of all cells descended therefrom.

## 2 Materials and methods

### 2.1 Ethical approval

Human tissue samples were obtained as part of the body donation program of the Institute of Anatomy, University Medical Center of the Johannes Gutenberg University Mainz, Mainz, Germany. The people donated their body voluntarily for medical education and research, and the present study was conducted within the parameters of the written permission we received from the body donor during lifetime. This research on human postmortem tissue was reviewed and approved by the Ethics Committee of Landesärztekammer Rheinland-Pfalz, Mainz, Germany (24/05/2022; Ref.# 2022-16488).

### 2.2 Sample preparation

The non-diseased brain and dermis samples of two (Hsa *n* = 2) male adult donors (dermis, prefrontal cortex, hippocampus, calcarine fissure, olfactory bulb, and cerebellum) were provided by the Institute of Anatomy, University Medical Center of the Johannes Gutenberg University, Mainz. The tissue samples were immediately snap-frozen after dissection and stored at –80°C until further processing.

### 2.3 DNA isolation

To isolate DNA from brain regions as well as dermis, the frozen tissues were cut under cooled conditions and aliquots of 20–30 mg were prepared. The genomic DNA of brain samples was isolated utilizing the QIAamp^®^ DNA Mini Kit, following the procedures of “Protocol: DNA Purification from Tissues (QIAamp DNA Mini Kit).”.

### 2.4 Fragmentation of genomic DNA

Fragmentation of 1 µg genomic DNA of brain samples (tester) and dermis sample (driver) was carried out with MboI (NEB) according to manufacturer’s protocol for 1 h at 37°C. Fragmented DNA was purified using phenol/chloroform/isoamyl alcohol (25:24:1), precipitated with ethanol, and resuspended in nuclease-free water.

### 2.5 Flank adapter ligation

Primers 5′-gca​gaa​gac​ggc​ata​cga​gat​ggc​att​ccg​gtc​t-3′ and 5′-gat​cag​acc​gga​atg​cc-3′ were hybridized by mixing 5 µL (100 pmol/μL) of each primer with 5 µL 5 M NaCl, heating the mixture to 100°C, and gradually cooling down to room temperature to allow annealing. Since the double-stranded adapter contains a “GATC”-overhang, ligation to 1 µg MboI-fragmented DNA can be carried out with NEBs T4 DNA ligase according to manufacturer’s protocol with 2 µL adapter solution overnight at 16°C. After ligation, excessive adapter was removed with Amicon^®^ Ultra 0.5 mL Centrifugal Filters (3 K).

### 2.6 Target site PCR

PCR amplification of 5′-flanking SVA regions of 50 ng ligated DNA was carried out with 10 pmoles primer 5′-gca​gaa​gac​ggc​ata​cga​gat-3′ (adapter complementary) and SVA consensus outward primer 5′-aga​atc​agg​cag​gga​ggt​tg-3′ according to the standard Qiagen Taq PCR core reaction, and the details of the thermal profile were as follows: 94°C for 120 s, 30 cycles amplification with 94°C for 40 s, 59°C for 40 s, and 72°C for 60 s and a final elongation at 72°C for 300 s. The semi-nested PCR was performed with the aforementioned thermal profile but amplified for 25 cycles and with the addition of 10 pmoles primer 5′-gca​gaa​gac​ggc​ata​cga​gat-3′ and 5′-atctgtgatcagtacmgtccagcttcggct-3, which introduces a new MboI restriction site at the SVA outward region of the PCR product.

### 2.7 RDA adapter ligation

PCR adapters of the tester (brain) and driver (dermis) PCR products were excised with MboI (NEB) at both ends according to manufacturer’s protocol and removed using Amicon^®^ Ultra 0.5 mL Centrifugal Filters (3 K) to finally introduce the new RDA adapters. RDA adapters 5′-acc​gac​gtc​gac​tat​cca​tga​acg-3′ and 5′-gatccgttcatg-3′ were hybridized and ligated, only to the tester sample and not driver, as described in Section 2.5. Finally, the samples were purified using Amicon^®^ Ultra 0.5 mL Centrifugal Filters (3 K).

### 2.8 Hybridization of the tester and driver

A 1:100 molar ratio of the tester (50 ng) and driver (5 µg) was set up for hybridization by mixing a total volume of 11 µL DNA, 2 µL 10 mM Tris (pH = 7.9), 1 µL 500 mM EDTA (pH = 8), and 2 µL nuclease-free water. The DNA mix and 5 M NaCl were preheated separately at 95°C for 2 min and subsequently 4 µL of 5 M NaCl was added to the DNA. The samples were covered with 30 µL mineral oil, denatured at 95°C for 4 min, and hybridized at 67°C for at least 18 h.

### 2.9 RDA PCR

Hybridized samples (250 ng) were prepared according to the standard Qiagen Taq PCR core reaction without the addition of primers and incubated at 72°C for 20 min to fill in overhangs after hybridization. After the addition of primer (10 pmoles) 5′-acc​gac​gtc​gac​tat​cca​tga​acg-3′ to the sample, amplification was carried out with the following thermal profile: 15 cycles amplification with 94°C for 40 s, 60.9°C for 40 s, and 72°C for 60 s and a final elongation at 72°C for 300 s. The samples (2 µL of the PCR product) were reamplified with the standard PCR reaction and primer (10 pmoles) 5′-acc​gac​gtc​gac​tat​cca​tga​acg-3′, and the details of the thermal profile were as follows: 94°C for 120 s, 20 cycles amplification with 94°C for 40 s, 60.9°C for 40 s, and 72°C for 60 s and a final elongation at 72°C for 300 s. Finally, excessive primers in the PCR product samples were eliminated with the addition of exonuclease I (NEB) according to manufacturer’s protocol.

### 2.10 Sequencing

PCR product sequencing on the Illumina NovaSeq 6000 platform with the 150 paired-end strategy was performed by Novogene Co., Ltd., and resulting raw data were provided as FASTQ files.

### 2.11 Bioinformatical scanning of somatic *de novo* SVA positions

Paired-end sequencing reads were merged using PEAR v0.9.6 (Zhang et al., 2014) and scanned for RDA primers. All reads containing the adapter were scanned for the SVA characteristic (CCCTCT)_n_ hexamer repeat and extracted. Reads containing a “GATC” restriction site between the genomic flank region and SVA part were eliminated as artefacts, since re-ligation of SVAs with a genomic fragment would be considered false *de novo* insertion. Hence, only reads with SVA sequences directly flanked by a genomic region were further processed, and the SVA parts were eliminated starting at the (CCCTCT)_n_ hexamer repeat. The remaining clean flank sequences were collapsed, length-filtered (only extract reads >30 bp) using SeqKit version 2.0.0 (Shen et al., 2016), and mapped to all GRCh38-annotated SVA sequences with 1 kb flanking regions, obtained by the UCSC Table Browser (https://genome.ucsc.edu/cgi-bin/hgTables; accessed on 06 January 2022) with the RepeatMasker track (Karolchik et al., 2004; Smit and Hubley, 2008; Smit et al., 2013), using BLAT v. 36 as the mapping tool (Kent, 2002). Reads that were mapped to SVAs or their respective flanking region were discarded, and the remaining reads were mapped to GRCh38 with BLAT v. 36. Only mapped reads with one chromosomal alignment were accepted, and to increase stringency, the obtained chromosomal positions were intersected with chromosomal positions of GRCh38-annotated SVA sequences with 3 kb flank using BEDTools v2.30.0 (Quinlan and Hall, 2010). Chromosomal positions that did not show shared positions with SVA plus flank were accepted as *de novo* somatic SVA positions. *De novo* SVA positions of one sample were extended to a broader chromosomal position when they overlapped with each other; thus, shared/intersecting chromosomal positions were “collapsed" to represent a single SVA integration event (chromosomal coordinates are provided in Supplementary Table S1).

### 2.12 Shared SVA position analysis

All *de novo* SVA positions of all brain regions from one person were combined to generate an individual reference BED file. Each brain region’s SVA positions were intersected with the reference file using BEDTools v2.30.0 and denoted as 0 (no overlap of reference position with the analyzed brain region) or >0 (overlap of reference position with a position of the analyzed brain region) to generate a presence/absence list for each brain region, respectively. Hence, each brain area receives a list of all SVA reference positions with character states as: position is present or absent. This list could be converted to a sequence, where one base like “A” is denoted as SVA position present and “T” as absent. The resulting sequences for each brain region were used to generate a maximum parsimony tree with branch lengths as steps and bootstrap resampling (1,000 replicates) utilizing MEGA11 (version 11) (Stecher et al., 2020; Tamura et al., 2021). Additionally, SeaView 4.0 was used to depict informative sites and bootstrap support with 1,000 bootstrap replicates (Galtier et al., 1996; Gouy et al., 2010).

To visualize shared SVA positions between brain regions, Venn diagrams were created using Intervene v0.6.5 (Khan and Mathelier, 2017).

### 2.13 Chromosomal SVA density analysis

The “summary” function in BEDTools v2.30.0 was used to estimate chromosomal SVA densities. To this end, chromosomal density of hg38-annotated SVAs was calculated as reference by estimating the average genomic SVA length using hg38 reference positions (UCSC Table Browser), multiplying SVA counts of each chromosome by the average SVA length, and dividing values by the chromosome size. This procedure was also utilized for each brain region by multiplying the SVA counts by hg38-estimated average SVA length. The arithmetic mean of *de novo* SVA density values for each chromosome was calculated based on the two donors (*n* = 2), and the correlation coefficient r, as a relation of *de novo* SVA density with hg38 SVA density, was calculated using the “CORREL (array1, array2)” function in Microsoft^®^ Excel v16.70. The *p*-value of correlation interpretation was calculated with the two-tailed Student’s t-distribution “T.DIST.2T” function.

### 2.14 Genomic feature and Gene Ontology analysis

The *de novo* SVA positions of each brain region in the BED format were used to annotate the related genomic feature using Homer v4.11 with the “annotatePeaks.pl” function (Heinz et al., 2010) and hg38 as reference. Additionally, Homer-annotated genes were analyzed using Metascape v3.5.20230101 (Zhou et al., 2019) for contribution to Gene Ontology pathways.

To validate the number of SVA integrations in SINE/LINE-rich regions, the hg38 reference genome was divided into 100 kb windows with a 10 kb stagger, and all LINE and SINE occurrences retrieved from the UCSC Table Browser with the RepeatMasker track were counted for each window. The average occurrence of combined SINE and LINE was calculated for all windows and set as a threshold, so that only windows exceeding the average were accepted as TE-enriched. In addition, only the top 25% TE-enriched windows were used as datasets. Both datasets containing the window coordinates were intersected with the *de novo* SVA insertion coordinates, which were annotated using HOMER as LINE- or SINE-associated, using BEDTools v2.30.0.

SVA flanks were extended up- and downstream by 2.5 kb, and the corresponding sequences were obtained from hg38 as reference using the BEDTools function “getfasta”. The GC content of the extended flanks was calculated, and the null hypothesis for consistency of the average GC content of flanks and genomic average GC content (hg38 = 40.9%) was tested with two-tailed Student’s t-distribution.

## 3 Results

### 3.1 SVA retrotransposition is active in the human brain and generates somatic mosaicism

**FIGURE 2:**
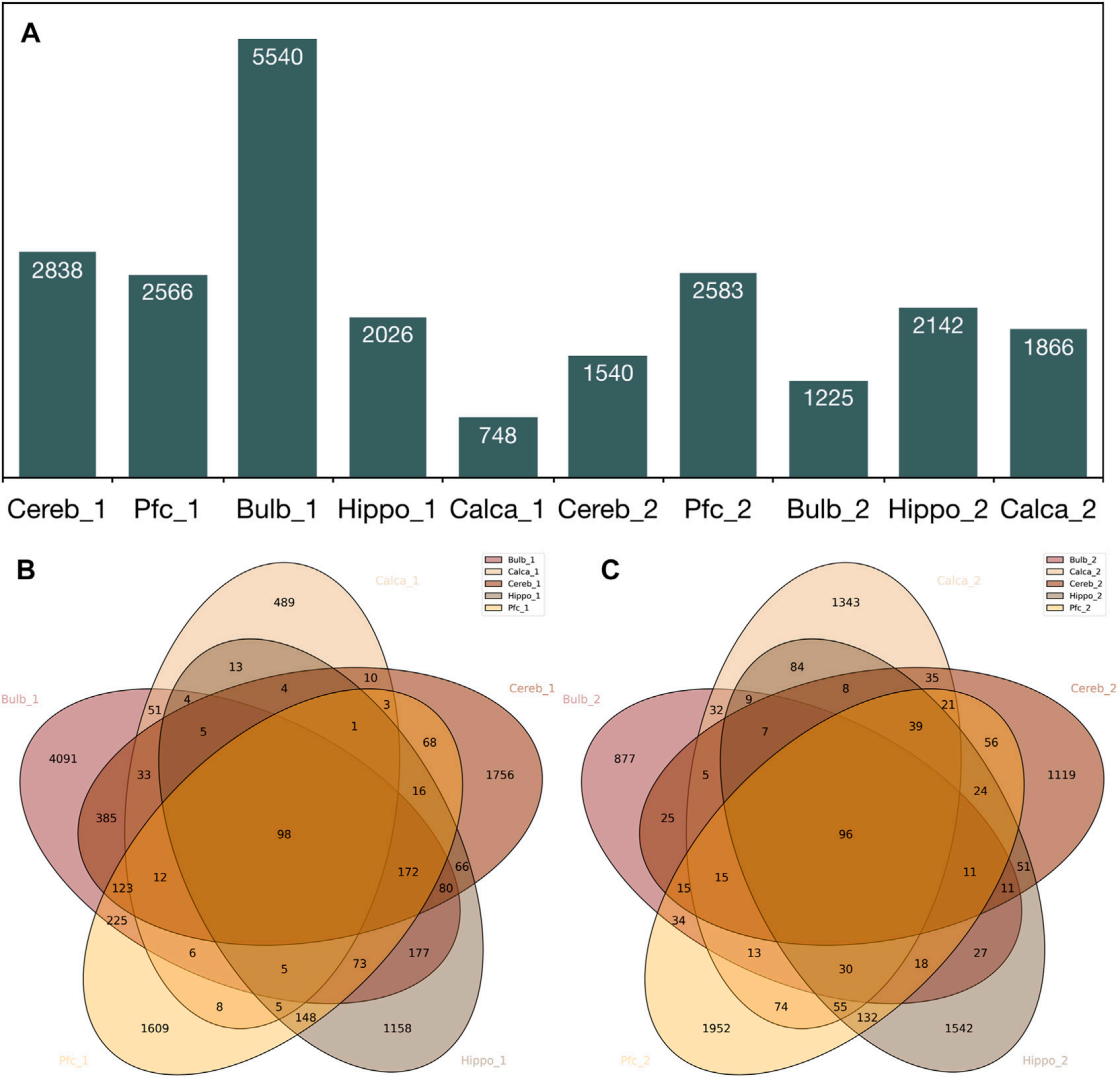
**(A)** Number of *de novo* SVA integrations for cerebellum (Cereb), prefrontal cortex (Pfc), olfactory bulb (Bulb), hippocampus (Hippo), and the calcarine sulcus (Calca); donors 1 and 2 are denoted as *_1 and *_2. **(B,C)** Venn diagrams of shared SVA integrations for brain regions depicted for both tested individuals.

The RDA method was applied to enrich unique 5′-SVA-flank templates, precisely *de novo* SVA insertions in brain regions, with template DNA from the dermis of the same individual as the driver sample. This driver sample represents the bulk of 5′-flanks for the germline-transmitted SVAs, both polymorphic and fixated SVA-integrations, and is given in a 100-fold molar excess to the RDA primer-ligated tester during hybridization. Since only tester-sequences are covalently ligated to RDA primers, SVA flanks that are not present in the driver were PCR-enriched, and the RDA-ligated sequences were extracted from the NGS output. As a result, we obtained somatic “SVA fingerprints” for the human brains of two male adult donors. We bioinformatically eliminated all annotated SVA portions of the collapsed reads and mapped the dataset of experimentally enriched SVA flanking sequences to the human genome (GRCh38), thus obtaining the coordinates of SVA insertions. In addition to the experimental reduction of germline SVAs by the RDA-implemented individual germline background (dermis), we eliminated the germline-transmitted SVAs annotated in hg38 with BED files of the respective coordinates. This resulted in the detection of 748–5,540 *de novo* SVA insertions in brain regions including the cerebellum (Cereb), prefrontal cortex (Pfc), olfactory bulb (Bulb), hippocampus (Hippo), and calcarine fissure (Calca) (Figure 2A). With an overall average of 2,307.4 (SEM = 413.22) *de novo* SVA positions, our findings provide ample evidence of active SVA retrotransposition in the human soma. Moreover, the result of SVA mobility rate in the brain is comparable to estimations of another study that counted 1,350 somatic SVA insertions in samples from the hippocampus and caudate nucleus as obtained by using a transposon capture method (Baillie et al., 2011). Next, we tested whether we were able to efficiently reduce the detection of SVA insertions potentially attributable to germline retrotranspositions by the enrichment procedures we applied experimentally. To this end, we counted putative *de novo* SVA insertions that coalesce deeply in ontogenesis and are therefore shared in all tested brain regions, meaning that they could represent the potentially germline-transmitted background of the SVA landscape. As a result, for the two individuals, we could only pinpoint 96 and 98 SVA deeply coalescing integrations, respectively (Figures 2B, C), accounting for only a small fraction of each individual’s SVA landscape. In fact, the majority of *de novo* insertions are traceable to take place on the lineages leading to telencephalon and metencephalon, since most of the examined somatic *de novo* SVA integrations are unique to different brain regions under scrutiny (Figures 2B, C). To count the number of reads supporting a *de novo* SVA insertion in the brain region-specific datasets, we initially collapsed the NGS-output after bioinformatically determining the flanks to datasets of non-redundant unique flanking sequences. When counting the non-redundant flanks specific for every unique *de novo* insertion, approximately 75–79% of *de novo* insertions were read one time (Supplementary Table S2; Supplementary Figure S1).

### 3.2 Somatic SVAs recapitulate general evo-devo patterns

**FIGURE 3:**
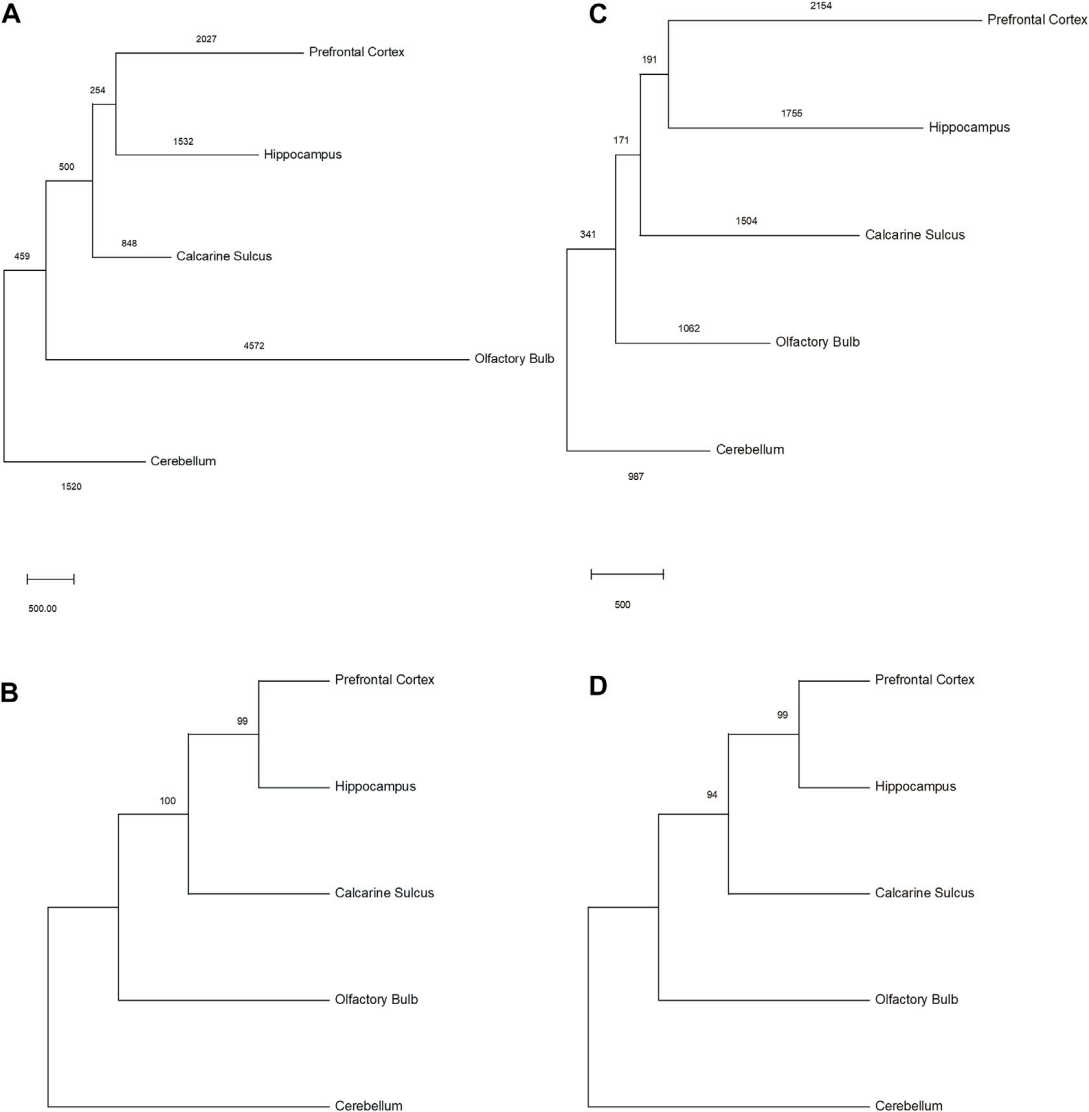
A maximum parsimony tree was constructed to phylogenetically relate the lineages, leading to brain regions based on *de novo* SVA positions. **(A)** Phylogenetic tree of donor 1 brain regions with values indicating branch length as steps (tree length = 11,710 steps) and **(B)** bootstrap support values of the phylogenetic tree (1,000 replicates). **(C)** Phylogenetic tree of donor 2 brain regions with values indicating branch length as steps (tree length = 8,163 steps) and **(D)** bootstrap support values of the phylogenetic tree (1,000 replicates).

For each individual, all existing *de novo* chromosomal SVA coordinates were combined as the reference file, de-duplicated, and intersected with the SVA *de novo* integration positions of each brain area to generate a presence/absence matrix. We applied an approach in which only overlapping *de novo* SVA positions of multiple brain regions are considered as shared integration, resulting in 1,775 (donor 1) and 918 (donor 2) *de novo* SVA insertions being present in more than one brain region of the tested individuals. Both unique and shared SVA integrations were used to generate a character-based data matrix with the presence/absence markers of SVA as character states to construct a maximum parsimony phylogeny of different telencephalic brain regions (Figure 3). The cerebellar dataset was set as an outgroup because this region is developmentally separate from the telencephalon as part of the metencephalon. In both individuals, the same phylogenetic branching patterns were generated and are well supported by the datasets as mirrored in the bootstrap values (Figures 3B, D). To obtain circumstantial evidence on the number of lineage-specific *de novo* integrations, a phylogenetic reconstruction without resampling was carried out. Altogether 10,888 characters or individual SVA integrations for person 1 and 7,757 for person 2 were analyzed, of which 1,487 and 717 were informative (Supplementary Table S2; Supplementary Figure S2), respectively. Non-informative were all integrations that occurred in one lineage only or are shared between all five brain regions. The respective phylogeny recapitulates the accepted ontogenetic processes of the brain with the longest branches leading to the ‘terminal taxa’ or brain regions (Figures 3A, C). As previously mentioned, the majority of SVA insertions remain unique to each brain region (Figures 2B, C). At this point, it should be noted that the net length of these edges can be understood as the sum of individual integrations that arose in the bulk of cells, from which DNA was prepared. This argues for extensive somatic SVA mosaicism in the adult human brain, even though in most cases, only a small number of unique integrations occur per cell. Although the internal branches are short, the number of shared integrations that coalesce on a retrotransposition taking place on the lineage leading to the common ancestor of the respective brain region-specific cells strongly supports the presented topology. Moreover, this well-supported topology was generated from two independent datasets.

From an anatomical and ontogenic perspective, the phylogenetic trees correspond well to the ontogenic and phylogenic origin of different brain regions. The cerebellum is part of the rhombencephalon, the third brain vesicle. The rhombencephalon divides into the metencephalon, which is the origin of the pons and cerebellum, and the myelencephalon, which is the precursor of the medulla oblongata. All remaining brain structures in the phylogenetic trees derive from the prosencephalon, the first brain vesicle. The prosencephalon divides into the telencephalon and diencephalon. Evolutionarily, the oldest structure of the telencephalon is the rhinencephalon (olfactory brain). The olfactory bulb derives from this ancient part of the cerebral cortex (paleocortex), and thus the distinctive separation may describe the early branching in the phylogenetic tree compared to the hippocampus as part of the archicortex and the prefrontal cortex and area striata as neocortical structures. The archicortex develops earlier than the neocortex and the localization of the area striata, respectively, calcarine sulcus, in the phylogenetic tree might be explained by the high specialization of this cortical region (granular cortex), which is important for the perception of visual stimuli.

### 3.3 Target regions of *de novo* SVA integrations

**FIGURE 4:**
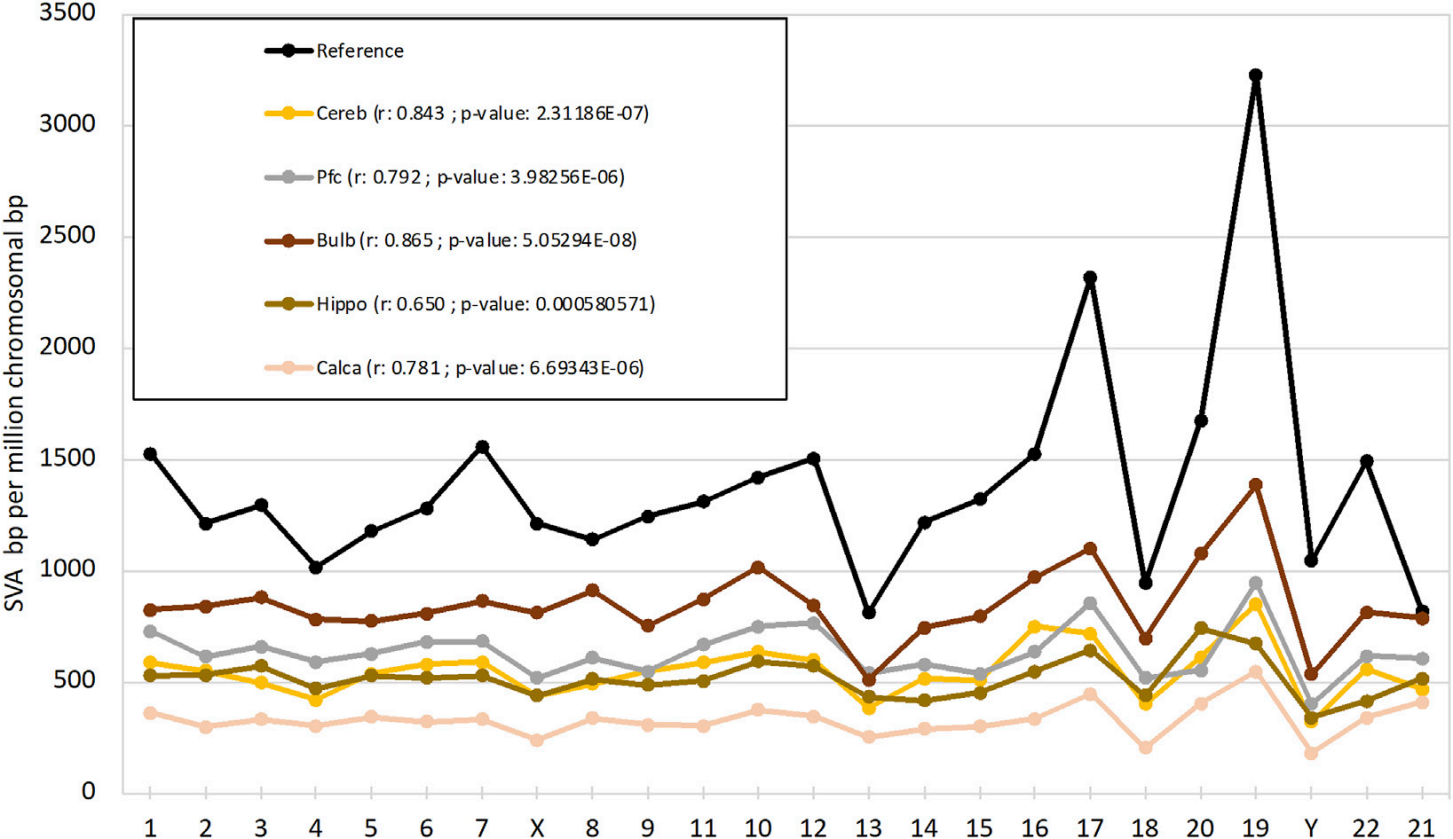
Chromosomal SVA density as SVA bp per million chromosomal bp on *y*-axis for reference SVAs (hg38) and *de novo* SVA integrations of each tested brain region (density values are calculated as mean values, *n* = 2). Human chromosomes are listed on *x*-axis. R-values and significance, depicted as *p*-values, indicate correlation of SVA density of reference with brain samples.

To compare our ontogenetic data in SVA integration targets with the targets that emerged over evolutionary timescales, we compared the presented *de novo* SVA integration datasets with germline SVA integration coordinates, as depicted in hg38. First, the *de novo* SVA density of each chromosome was estimated by calculating the average genomic SVA length of hg38, which was then multiplied by the SVA counts of each chromosome and finally corrected for chromosome size. The resulting density values are depicted in Figure 4 as the arithmetic mean (*n* = 2) of SVA bp per million chromosomal bp for each brain region and chromosome. The reference, based on hg38-annotated SVA data, and *de novo* SVA positions in each brain region recapitulate general evolutionary patterns of chromosome-specific SVA density and reveal chromosome-wide rates of *de novo* SVA retrotranspositions. Here, the detected preferences for specific chromosomes, such as Chr. 17 and Chr. 19, are largely consistent with evolutionarily conserved chromosomal SVA patterns.

Concordantly, other reports also found SVA elements to be more frequent than those expected on chromosome 17 and especially chromosome 19, whereas chromosomes 13, 18, and Y exhibited less targets for the reintegration of SVAs (Wang et al., 2005; Tang et al., 2018). Taken together for all brain regions under scrutiny, the correlation coefficient r indicates a strong positive correlation of SVA density of reference with all tested brain regions (*p* < 0.05), suggesting similar upward and downward trends of SVA densities on human chromosomes with preferential integration regions.

**FIGURE 5:**
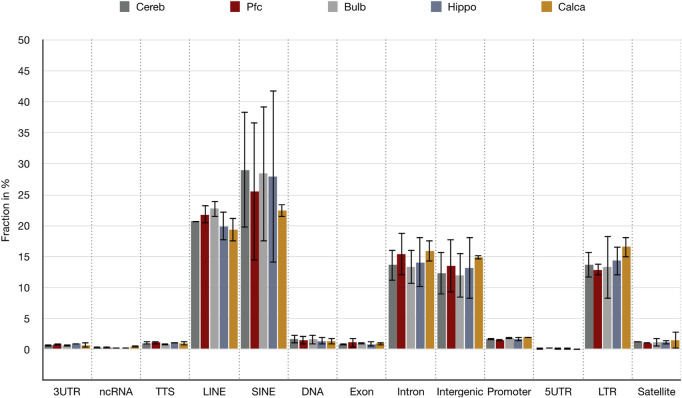
Annotation of genomic features of *de novo* SVA integrations for each brain region. Mean values (*n* = 2) of fractions of annotated features with respect to sum of all HOMER-annotated features are displayed in % with the standard deviation shown as bars. Features are: 3′-untranslated region (3′-UTR), non-coding RNA (ncRNA), transcription termination site from −100 bp to +1 kbp (TTS), LINE transposons (LINE), SINE transposons (SINE), DNA transposons (DNA), exonic region (Exon), intronic region (Intron), intergenic region (Intergenic), promoter-TSS from −1 kbp to +100 bp (Promoter), 5′-untranslated region (5′-UTR), long terminal repeats (LTR), and satellite region (Satellite).

To quantify a possible enrichment of *de novo* SVA integrations in sites with defined genome features, we applied HOMER software with the *de novo* SVA integration coordinates. In this way, the genomic features of all *de novo* SVA positions were annotated and displayed as fractions of the total annotated features for each brain region (Figure 5). Interestingly, the SVA elements favored integration in genomic positions containing retrotransposon families of LINEs, LTRs, and short interspersed nuclear elements (SINEs), more precisely Alus, as well as intronic and intergenic regions. We checked whether the regions with LINE or SINE association of *de novo* SVA insertions are generally TE-rich regions. To that end, we divided the human genome in 100 kb windows with a 10 kb stagger, extracted all hg38-annotated LINE and SINE positions, and counted the occurrences, i.e., the sum of SINEs and LINEs, for each window. The average count of retrotransposition events within the windows was set as the normal density of TEs, and all windows above average were extracted as TE-rich windows. In addition and for more stringent analysis, we used only the top 25% TE-enriched windows (highest LINE/SINE count windows). We then intersected the two datasets containing the window coordinates with *de novo* SVA integration positions that are associated with SINE or LINE sequences according to the HOMER annotation; 72.83–92.23% of LINE- or SINE-associated *de novo* SVA integrations are located in 100 kb windows with higher SINE/LINE count than that in average 100 kb windows (Table 1). When only the top 25% TE-rich 100 kb windows are considered, 51.25–62.08% of *de novo* SVAs are still located within the TE-enriched region. In conclusion, *de novo* SVA integrations tend to fall in regions with high count of both SINE and LINE families, which are enriched together. Therefore, *de novo* SVA integration is apparently preferred in regions where previous retrotranspositions occurred, such as HOMER-annotated LINE-2 families (Supplementary Table S2; Supplementary Figure S3), that are mostly truncated remnants mobilized before the mammalian radiation, or LINE-1 as the only remaining autonomous mobilizing element in humans (Zhang et al., 2020). In addition to integration near retrotransposon sequences, *de novo* SVA insertions associated with intronic and intergenic regions show that integrations preferentially occur at genomic loci where disruption of gene integrity is less likely.

**TABLE 1:** *De novo* SVA integrations associated with retrotransposon-rich regions.

Sample (donor 1 = *_1; donor 2 = *_2)	% of LINE/SINE-associated *de novo* SVA insertions in TE-rich 100 kb windows (average TE count as the threshold)	% of LINE/SINE-associated *de novo* SVA insertions in TE-rich 100 kb windows (top 25% TE-enriched windows)
Cerebellum_1	78.36	54.83
Prefrontal_cortex_1	88.24	62.08
Olfactory_bulb_1	80.11	53.35
Hippocampus_1	79.70	57.03
Calcarine_sulcus_1	86.71	58.86
Cerebellum_2	88.57	55.64
Prefrontal_cortex_2	90.16	52.19
Olfactory_bulb_2	72.83	51.25
Hippocampus_2	92.23	61.71
Calcarine_sulcus_2	82.05	57.35

From chromosome-specific SVA integration target densities and the genome feature analysis, we conclude that reintegrations of SVA target sites with similar characteristics, both in the germline and in the ectodermal brain cells. Therefore, it is reasonable to assume that this causes comparable regulatory consequences, suggesting similar retrotransposition modes being effective in both the germline and soma.

### 3.4 *De novo* SVA integrations frequently locate in close proximity to neural-specific genes

To elucidate the functional consequences of *de novo* integrations, we examined the genes that could be physically linked to the gene body features extracted from HOMER analysis. To this end, we first removed the intergenic regions that were physically close to *de novo* SVA integrations. We then focused on the related gene names of annotated genomic features linked to *de novo* integrated SVAs like promoters, introns, exons, and 5′-and 3′-UTRs (Figure 5). The gene names were subsequently analyzed for their association using Gene Ontology analysis (Supplementary Table S3). We found that genes physically close to certain somatic SVA integrations fall into neural-specific Gene Ontology pathways and the most significant pathways are linked to regulation of synapse structure or activity (GO:0050803), synaptic signaling (GO:0099536), neuronal system (R-HSA-112316), behavior (GO:0007610), nervous system development (R-HSA-9675108), and neuron projection morphogenesis (GO:0048812). This supports the assumption that SVA retrotranspositions, related to genes of neural-specific pathways, represent events within the lineages of the somatic brain, where neural-specific gene activity is linked to active chromatin states and provides conditions for active retrotransposition. Similar prevalent conditions, where SVA insertions are favored in active chromatin with genic regions, were also reported by Savage et al. (2013).

Since gene density correlates with GC density (Lander et al., 2001; Versteeg et al., 2003) and SVAs can insert in proximity to genes (Figure 5), we determined whether SVA *de novo* insertions are established in GC-rich regions as detected by Raiz et al. (2012) in 5-kb- and 30-kb-long SVA flanking regions. To this end, we calculated the average GC content of 5-kb extended SVA flanks for each brain region (Table 2) and compared the values to the average GC content of the genome hg38. With an average of 42.82% GC in all tested brain samples, the GC content was tested to be significantly different (*p* < 0.05) from the 40.9% genome average (Piovesan et al., 2019). Consequently, with an approximately 2% increase in the GC content, *de novo* SVA insertions tend to prefer GC-rich regions over AT-rich regions.

**TABLE 2:** Average GC content of all SVA flanks (5 kb extended) for each tested sample.

Sample (donor 1 = *_1; donor 2 = *_2)	Average GC content of all SVA flanks (%)	*p*-value (testing H_0_: GC content of SVA flanks and genome hg38 are consistent)
Cerebellum_1	42.94	6.85205E-73
Prefrontal_cortex_1	43.06	2.4634E-72
Olfactory_bulb_1	42.79	1.3077E-126
Hippocampus_1	42.89	2.73278E-50
Calcarine_sulcus_1	42.99	6.44965E-22
Cerebellum_2	42.82	7.9799E-35
Prefrontal_cortex_2	42.24	3.57767E-29
Olfactory_bulb_2	42.80	2.46387E-29
Hippocampus_2	42.51	2.02962E-35
Calcarine_sulcus_2	43.18	1.12244E-55

## 4 Discussion

The proposed method of RDA-implemented enrichment of *de novo* SVA insertions provides further evidence of active SVA retrotransposition in the human brain. We were able to detect 748 somatic SVA insertions in the calcarine sulcus to 5,540 in the olfactory bulb. Based on the primer system adopted from the ME-Scan-SVA method (Ha et al., 2016) and the authors’ estimation of the fractions of different SVA families that could be amplified with these primers, we believe that our results are composed of conservative estimations. The quantitative estimations of *de novo* SVAs in our enrichment method are in the same range as those proposed by applying other capture methods, for example, demonstrated by Baillie et al. (2011). In contrast to other methods, RDA focuses on rare genomic changes and utilizes informative clade markers with distinct character polarity as indicators of the frequency of independent insertions. With the RDA-implemented technical reduction of the individual SVA background (driver = same individual dermis) and by excluding hg38-annotated SVAs as a bioinformatical reduction of germline-transmitted SVAs, we were able to decrease the detection of potential *de novo* SVA insertions attributable to retrotranspositions in the germline or outside the brain during early embryogenesis. The result is that only 96 and 98 SVA positions occur in all tested brain regions, thus representing only a small portion of each person’s SVAs. Additionally, we report 1,775 (donor 1) and 918 (donor 2) shared *de novo* SVA insertions in more than one brain region of the tested individuals, with prefrontal cortex, hippocampus, and calcarine fissure being grouped as regions with the highest similarity as obtained from phylogenetic analysis. Because the brain is among the organs that start to emerge early in prenatal development and among the last to complete postnatal development, genetic alterations such as SVA insertions are difficult to attribute to the developmental timing or progenitor cell population that contribute to the similarity of the aforementioned regions. Nonetheless, the majority of SVA insertions are unique to each brain region and thus can indeed be attributed to brain lineages, confirming the observation of distinct somatic mosaicism in the adult human brain in agreement with Baillie et al. (2011) and Evrony et al. (2015) who demonstrated active SVA, Alu, and L1 retrotransposition in the human brain. We hypothesize that the fact that the observed proportion of unique *de novo* integrations is high, could be explained by their preferential occurrence in many postmitotic neurons; thus the respective *de novo* integrations are not transmitted into progeny cells. In contrast, the smaller proportion of multiple-read *de novo* integrations might occur in mitotic brain cells, e.g., glial cells.

Although we can assign a unique SVA insertion to a specific brain area, such as the prefrontal cortex or hippocampus, our bulk DNA preparation does not allow further assignment to a defined neuronal cell type because we did not use a method for appropriate differentiation, such as cell sorting. Our motivation to start with bulk cell preparations of brain areas to detect *de novo* SVA insertions was based on the assumption that brain neurons and all resident cell types form a functional unit that contributes to a physiologically functional brain. Several brain diseases can be associated with pathological changes in specific cell types, such as interneurons and microglia, and autism, schizophrenia, and Alzheimer’s disease can be associated with changes in all major brain cell types (Skene and Grant, 2016). Thus, the demonstrated somatic mosaicism in the brain may have functional consequences for health and disease, regardless of the cell type.

We examined target region preferences of *de novo* SVA insertions at multiple levels, including the chromosomal location and gene features. First, we found that *de novo* integration preferentially targets transposon-rich regions. We demonstrated that *de novo* SVA insertions occur in regions with high numbers of L1, Alu, and LTRs. When comparing SVAs transmitted across evolutionary timescales, we find a striking similarity. Thus, both germline and somatic brain cells tend to have similar target regions in terms of frequencies of SINE/LINE families as annotated by HOMER analysis. This suggests that similar retrotransposition modes associated with the in trans effects of the autonomous mobilizing LINE-1 are operative in both the germline and soma. In addition, our *de novo* SVA density data suggest similarities with evolutionarily conserved SVA patterns, with chromosomes 17 and 19 showing higher SVA frequencies and chromosomes 13, 18, and Y showing lower SVA frequencies, comparable to our hg38 reference data and the reports of Tang et al. (2018) and Wang et al. (2005). Chromosome 19 appears to be particularly notable in terms of high SVA integration rates, thus confirming previous data from Grimwood et al. (2004) who described chromosome 19 as a chromosome with both high transposon content and gene density. Overall, SVA retrotransposition is thought to occur preferentially in gene-rich and active chromatin regions, as observed by Savage et al. (2013), reflecting the situation in the germline and providing ample opportunities to fine-tune gene expression patterns. Barnada et al. (2022) also reported that the epigenetic repression of active SVAs results in differential gene expression of genes near SVAs. Based on our HOMER results, we also detected *de novo* SVA positions near genes, particularly in association with intronic, promoter, and other gene-related regions. In addition, we were able to confirm the results of Barnada et al. (2022) showing the same mode of preferred retrotransposition in close proximity to gene bodies and that a fraction of the genes associated with these SVA positions can be assigned to neural-specific Gene Ontology pathways. Another result of our study shows that the intersection of the same target *de novo* integrations is low in the two individuals studied, and this shared portion could be the cause of probabilistic target region preferences, i.e., the frequency of SINEs/LINEs and neural genes that are more active with an open chromatin state in the human brain (Supplementary Table S2; Supplementary Figure S4). Finally, the GC content within the 5-kb flanking regions of *de novo* SVA insertions was higher than the average of the human genome, suggesting that SVAs in general tend to insert in genic and GC-rich regions, besides TE-rich regions, in agreement with the results of Raiz et al. (2012) and Wang et al. (2005).

To summarize, our data on somatic SVA mosaicism in the brain demonstrate the mobility of a class of retrotransposons that is highly mobile in the human germline, too. Moreover, there is a striking similarity of retrotransposition modes between the germline and soma, as suggested by similar target regions and gene regulatory potential. We hypothesize that transcribed brain genes trigger chromatin states to be amenable for retrotransposition, as suggested by the correlation of physical distances between brain gene loci as uncovered by GO analysis and somatic SVA integrations. Therefore, somatic mosaicism of SVAs in the human brain is of particular interest, since brain disorders such as Parkinson’s disease can be associated with the presence or absence of SVAs at orthologous loci, along with altered gene expression (Pfaff et al., 2021). We were able to obtain data on the level of multilocus SVA mobility in all tested brain areas, resulting in many lineage specific *de novo* SVA insertions that are frequently associated with genes in close proximity and thus possibly associated with differential gene expression as described by Pfaff et al. Moreover, we described *de novo* SVA insertions that take place at earlier stages of brain development in cells that are still mitotic, giving rise to cell lineages phylogenetically linked to the presence/absence of SVA clade markers. The temporally and spatially ubiquitous *de novo* SVA integrations in the brain could be used as clade markers to study the origin and evolution of brain tumors, that is, to reconstruct intratumor heterogeneity and the tumor cell lineages’ phylogeny. The proposed RDA–NGS method to define *de novo* SVA integrations is able to detect unique SVA integrations in tester as compared to driver genomes. Thus, the mutation catalog of a brain tumor can be supplemented with the non-reversible presence/absence of SVA markers at orthologous loci with that—besides obtaining information on tumor heterogeneity—tissue-specific tumor origin, lineages of cell populations harboring cancer promoting mutations, or primary sites of metastasis could be pinpointed. Furthermore, since there are some limitations in defining and naming cell types with dynamic markers, Domcke and Shendure (2023) recommended establishing a data-driven “consensus ontogeny” to differentiate cell lineages, e.g., in fetal hematopoiesis or intra- and inter-individual variations. As part of an attempt to order cells based on differences in molecular states and lineage history in a tree-based approach, SVAs as stable clade markers, together with their definition by RDA, could provide an additional tool in the field of cell lineage tracing.

## Data Availability

The datasets presented in this study can be found in online repositories. The names of the repository/repositories and accession number(s) can be found at: NCBI BioProject under PRJNA949405.
