# Supplementary material for: RDA coupled with deep sequencing detects somatic SVA-retrotranspositions and mosaicism in the human brain
Source: Front Cell Dev Biol. 2023 Jun 1;11:1201258. doi: 10.3389/fcell.2023.1201258 (PMC10267396; doi:10.3389/fcell.2023.1201258)
Supplement: Supplementary file 2 [file Table2.docx]

Supplementary Material

RDA coupled with deep sequencing detects somatic SVA-retrotranspositions and mosaicism in the human brain

Jonas Möhner^1*^, Maurice Scheuren^1^, Valentina Woronzow^1^, Sven Schumann^2^, Hans Zischler^1*^

*** Correspondence:** Jonas Möhner, Hans Zischler,
[moehner@uni-mainz.de](mailto:moehner@uni-mainz.de) , [zischler@uni-mainz.de](mailto:zischler@uni-mainz.de),

# Supplementary Figures


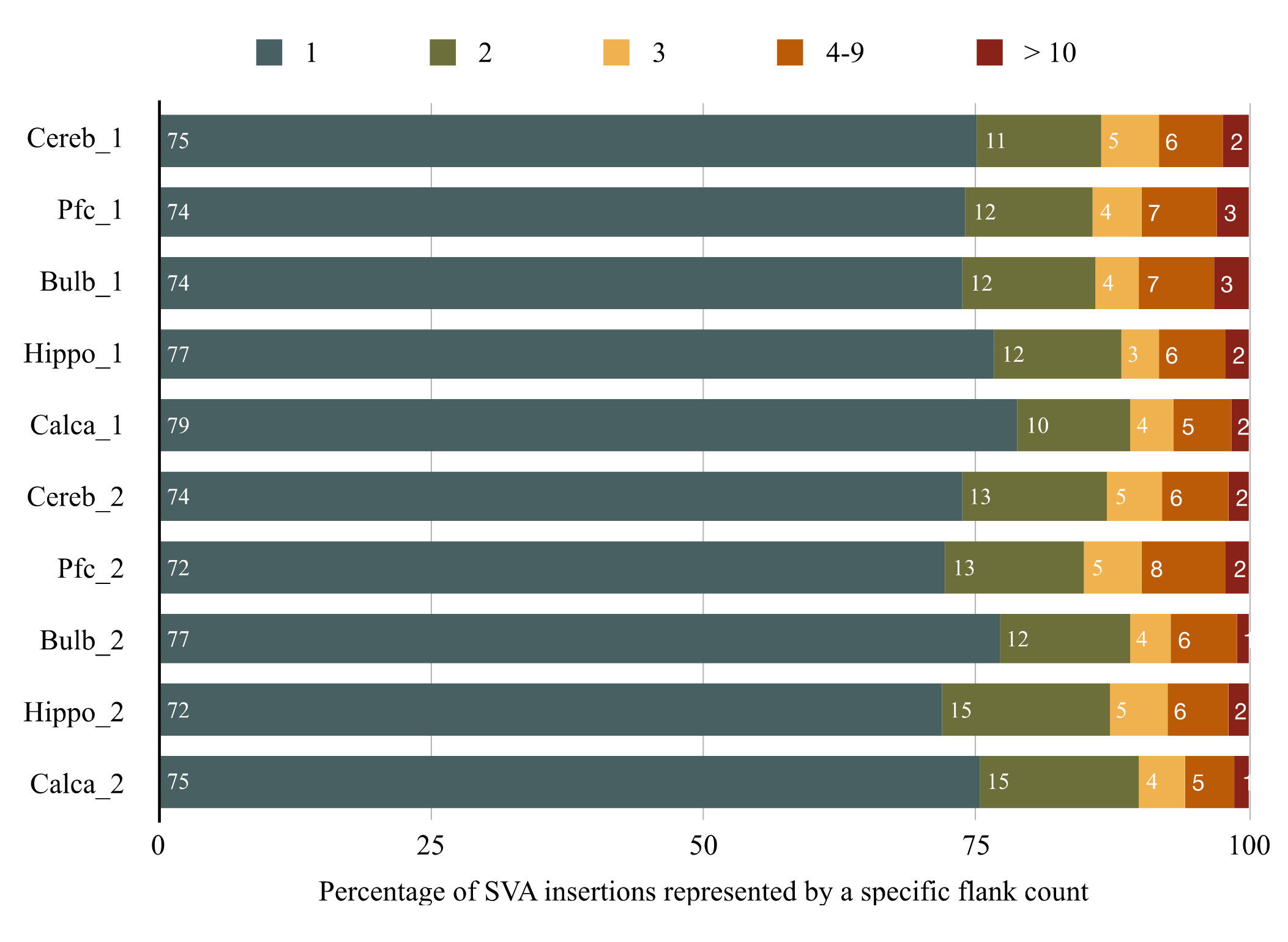


Supplementary Figure S1. Number of non-redundant flank counts attributed to each unique SVA insertion event. X-axis shows percent of SVA positions represented by 1, 2, 3, 4-9 or > 10 flank counts.


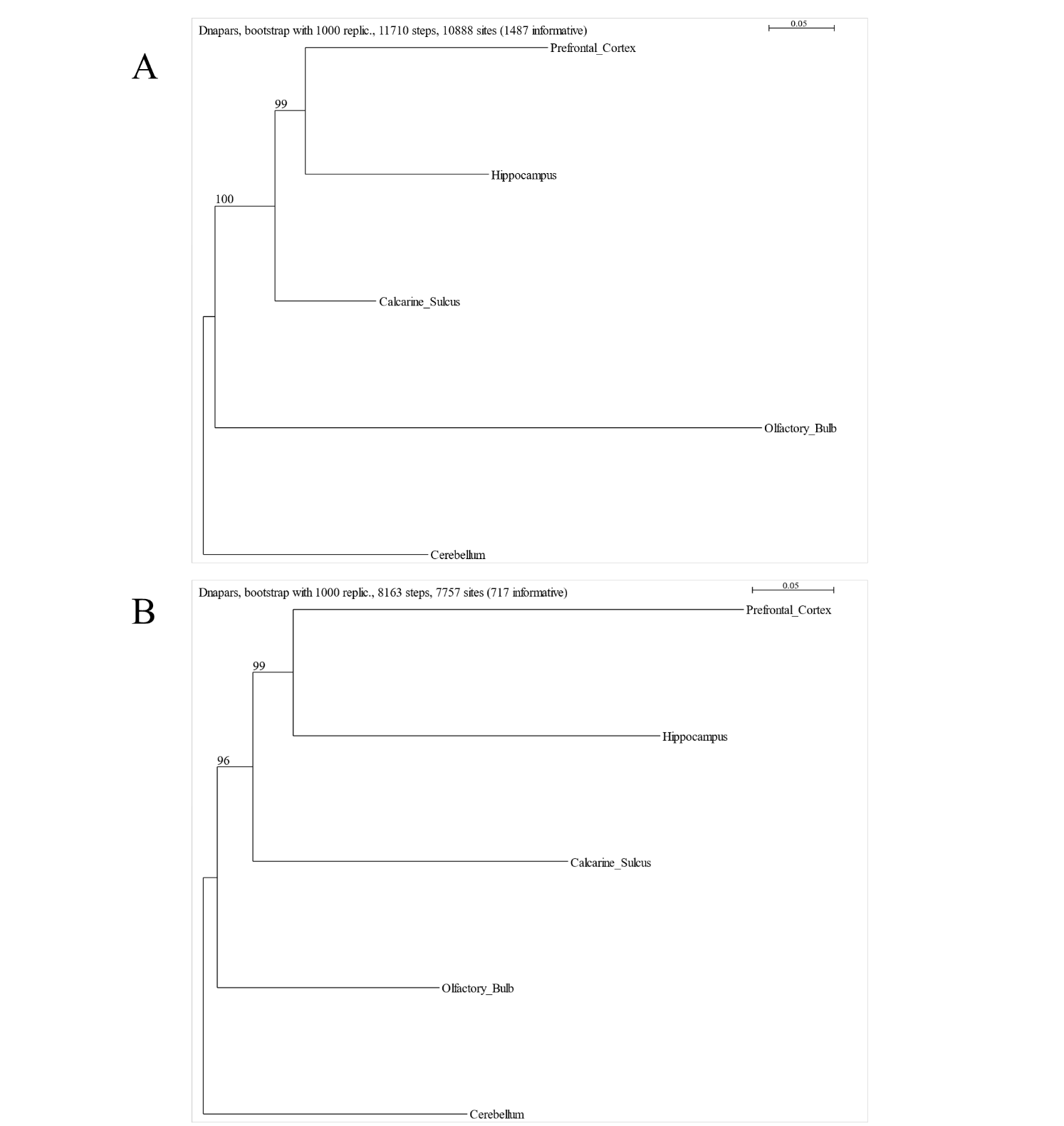


Supplementary Figure S2. Maximum parsimony tree for relation of tested brain regions in respect to de novo SVA positions, generated with SeaView 4.0. (A) Phylogeny of donor one brain regions and (B) donor two brain regions with bootstrap support values (1000 replicates).


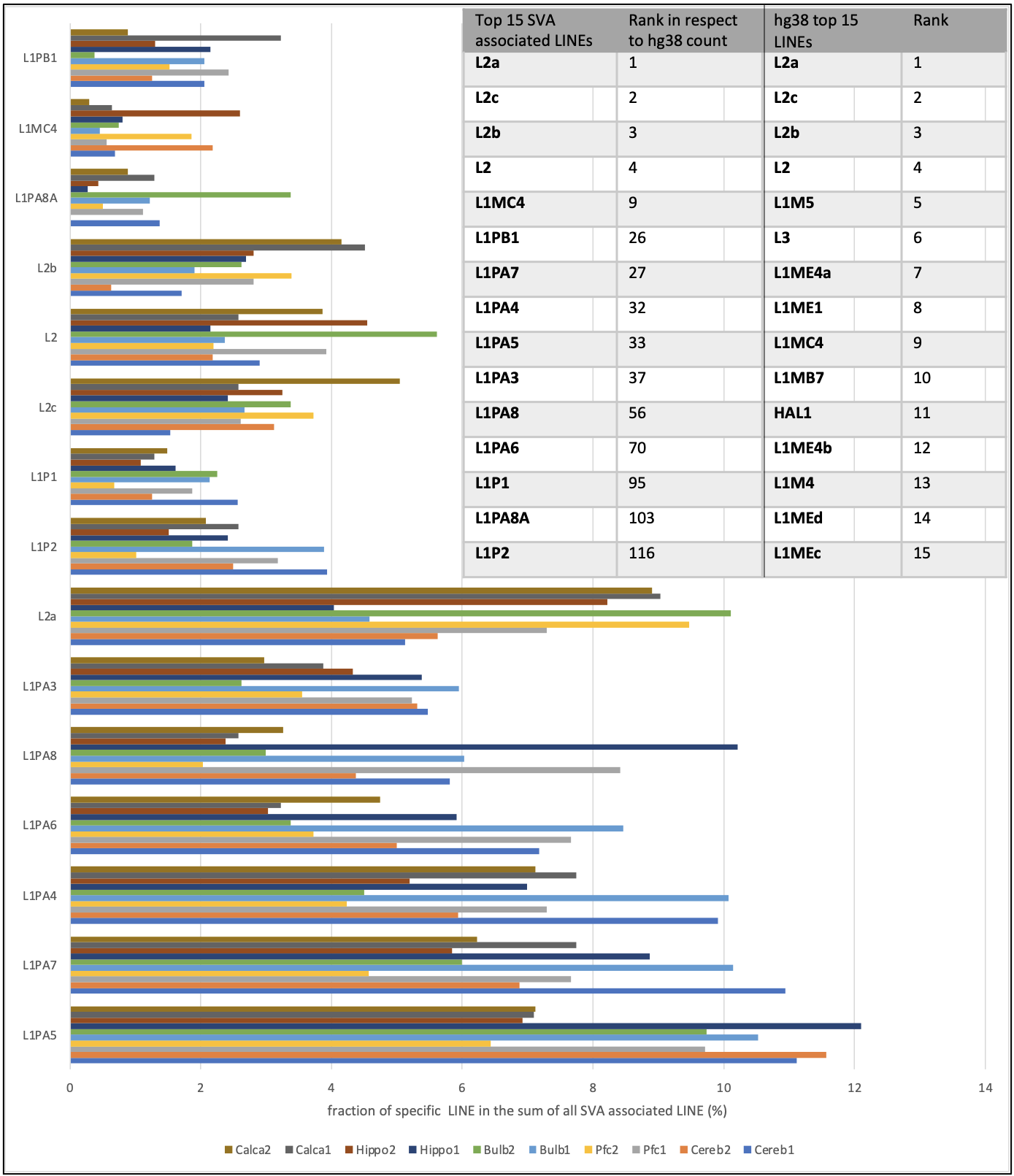


Supplementary Figure S3. Diagram of top 15 HOMER annotated LINE families at de novo SVA positions as fraction of sum of all HOMER annotated LINE families in %. The table in the upper right corner depicts the top 15 HOMER annotated LINEs in the first column and their ranking in the hg38 reference (absolute counts of each TE in hg38 as basis of ranking, e.g. 1 = highest occurrence) in the second column. Column 3 and 4 depict the top 15 LINEs in hg38.


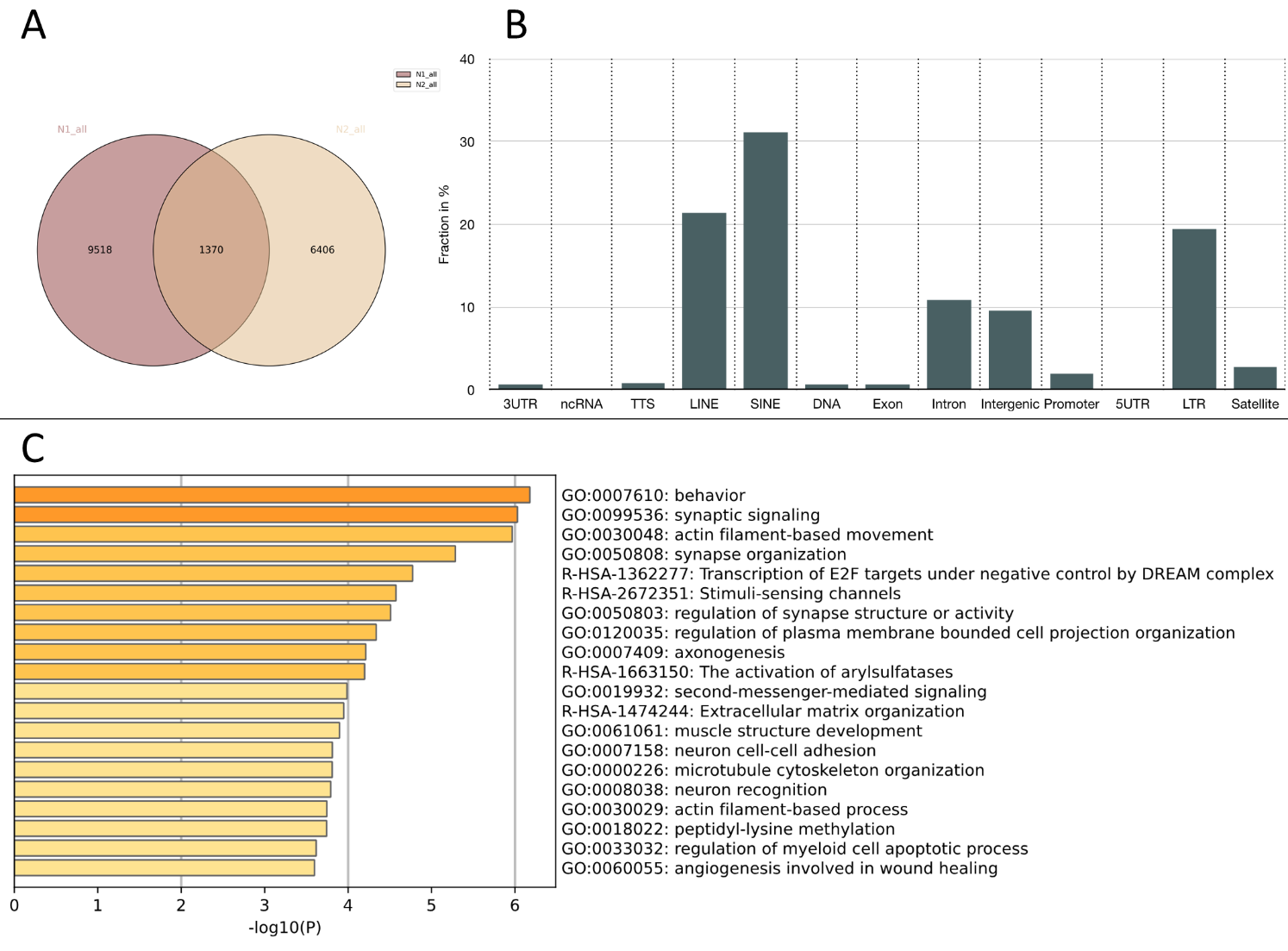


Supplementary Figure S4. (A) Venn diagram of shared de novo SVA positions of donor one (N1) and donor two (N2). (B) HOMER-annotation of genomic features of shared de novo SVA positions. Fraction of annotated feature with respect to sum of all annotated features are displayed in %. Features are: 3´ untranslated region (3UTR), non-coding RNA (ncRNA), transcription termination site from −100 bp to +1kbp (TTS), LINE transposons (LINE), SINE transposons (SINE), DNA transposons (DNA), exonic region (Exon), intronic region (Intron), intergenic region (Intergenic), promoter-TSS from −1kbp to +100 bp (Promoter), 5´ untranslated region (5UTR), long terminal repeats (LTR), satellite region (Satellite). (C) Metascape gene ontology pathways of genes in close proximity to SVA positions that are shared in both individuals.
